# Calibration of PET/CT scanners for multicenter studies on differentiated thyroid cancer with ^124^I

**DOI:** 10.1186/s13550-016-0191-x

**Published:** 2016-04-27

**Authors:** Jakob W. Kist, Manfred van der Vlies, Otto S. Hoekstra, Henri N. J. M. Greuter, Bart de Keizer, Marcel P. M. Stokkel, Wouter V. Vogel, Marc C. Huisman, Arthur van Lingen

**Affiliations:** Department of Nuclear Medicine, Netherlands Cancer Institute, Plesmanlaan 121, 1066 CX Amsterdam, The Netherlands; Department of Diagnostics, Groene Hart Ziekenhuis, Bleulandweg 10, 2803 HH Gouda, The Netherlands; Department of Radiology and Nuclear Medicine, VU University Medical Center, De Boelelaan 1117, 1081 HV Amsterdam, The Netherlands; Department of Nuclear Medicine, University Medical Center Utrecht, Heidelberglaan 100, 3584 CX Utrecht, The Netherlands

**Keywords:** Calibration, Multicenter, Quantification, PET/CT, Iodine-124, Differentiated thyroid cancer

## Abstract

**Background:**

Studies on imaging of differentiated thyroid cancer (DTC) using ^124^I often require a multicenter approach, as the prevalence of DTC is low. Calibration of participating scanners is required to obtain comparable quantification. As determination of a well-defined range of recovery coefficients is complicated for various reasons, a simpler approach based on the assumption that the iodine uptake is highly focal with a background that significantly lacks radioactivity might be more efficient. For each scanner, a linear conversion between known and observed activity can be derived, allowing quantification that can be traced to a common source for all scanners within one study-protocol. The aim of this paper is to outline a procedure using this approach in order to set up a multicenter calibration of PET/CT scanners for ^124^I.

**Methods:**

A cylindrical polyethylene phantom contained six 2-ml vials with reference activities of ~2, 10, 20, 100, 400, and 2000 kBq, produced by dilution from a known activity. The phantom was scanned twice on PET/CT scanners of participating centers within 1 week. For each scanner, the best proportional and linear fit between measured and known activities were derived and based on statistical analyses of the results of all scanners; it was determined which fit should be applied. In addition, a Bland-Altman analysis was done on calibrated activities with respect to reference activities to asses the relative precision of the scanners.

**Results:**

Nine Philips (vendor A) and nine Siemens (vendor B) PET/CT scanners were calibrated in a time period of 3 days before and after the reference time. No significant differences were detected between the two subsequent scans on any scanner. Six fitted intercepts of vendor A were significantly different from zero, so the linear model was used. Intercepts ranged from −8 to 26 kBq and slopes ranged from 0.80 to 0.98. Bland-Altman analysis of calibrated and reference activities showed that the relative error of calibrated activities was smaller than that of uncalibrated activities.

**Conclusions:**

A simplified multicenter calibration procedure for PET/CT scans that show highly focal uptake and negligible background is feasible and results in more precise quantification. Our procedure can be used in multicenter ^124^I PET scans focusing on (recurrent) DTC.

## Background

Iodine-124 (^124^I) is currently of great interest as a PET/CT tracer in patients with (metastasized) differentiated thyroid cancer (DTC) for pre-therapeutic assessment of iodine avidity of lesions and for dosimetric purposes [1–4]. Dosimetry requires reliable quantification, and as gathering strong clinical evidence in this relatively rare disease requires multicenter studies to ensure sufficient patient enrollment, calibration of scanners is required [5]. The concurrent emergence of the European Association of Nuclear Medicine (EANM) Research Ltd (EARL) accreditation procedure for 2-[18F]fluoro-2-deoxy-d-glucose (^18^F-FDG) imaging aims to achieve comparable scanner performances across multiple sites through harmonization of the acquisition of PET/CT scans [6]. However, in the case of ^124^I used in DTC patients, a standardization strategy as used for ^18^F-FDG is not adequate because recovery of the partial volume effect is difficult to determine [7]. Due to the combination of the low positron abundance (around 25 %) and the presence of 602-keV non-annihilation photons, image-derived activity concentrations are inaccurate [7, 8]. Documented recovery coefficients depend on object volume and shape, background activity, voxel size, and number of effective iterations [7]. Due to the high specificity of iodine for thyroid tissue, the uptake in the background is negligible. This allows to determine the total activity within the lesions by drawing an oversized volume of interest (VOI) around the imaged target, avoiding the unknown influence of the partial volume effect. The lesion uptake in units of activity concentration or standardized uptake value (SUV, %) can be calculated from the total activity and the lesion volume, determined from anatomical imaging, e.g., a CT scan.

By measuring a range of ^124^I activities in a phantom experiment, a linear relation between the reference and measured activities can be derived per scanner. Hence, measured activities can be converted to calibrated activities for all scanners used in a multicenter study. Knowledge of the inaccuracies of individual scanners allows for benchmarking and thereby determining underperforming scanners. Excluding these scanners will improve the overall accuracy of the quantification of ^124^I in a multicenter study and thereby its quality. The aim of this paper is to outline a procedure for multicenter calibration of the total activity of ^124^I in focal, low-background areas.

## Methods

**Fig. 1:**
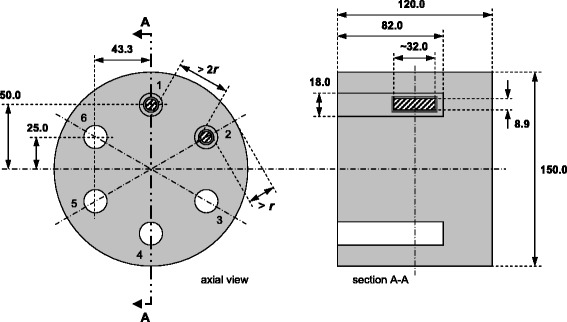
Design of the phantom. All sizes are in millimeters. The height is lower than the smallest axial field of view of the PET/CT scanners included for calibration. The phantom consists of solid polyethylene (*light gray*) and contains six openings (indicated 1 to 6; 3 to 6 *colored white*). In each opening, a polyethylene socket can be placed tightly, enclosing an amber glass vial (*dark gray*, wall thickness about 1.5 mm) of 2 mL inner volume, filled with ^124^I activity (*shaded*). The position of the vials in the phantom is such that the ^124^I positrons (maximum range *r*) do not interfere between the vials and do not reach the outside of the phantom. The vials have a neck and screw cap, so the shape and height are an indication

A cylindrical polyethylene phantom containing six 2-ml cylindrical glass vials (Fig. 1), representing typical lesion volumes, was developed in-house. By weighing and diluting from a known activity of ^124^I (BV Cyclotron, Amsterdam, The Netherlands), reference activities (*A*_r_) of approximately 2, 10, 20, 100, 400, and 2000 kBq at reference time (*T*_r_) were obtained and put into the vials. The activity-series was based on the Thyropet protocol, the optimized dosimetry protocol by Jentzen et al., and calculations with the iodine kinetic model described in ICRP publication 53 [3, 9–11]. No activity in the background was used. A non-radioactive solution of approximately 1 mg/mL iodine was used for dilution to prevent ^124^I from sticking to the walls. Reference activities at the time of calibration of the scanners (*T*_c_) were obtained by correction for decay of ^124^I (half-life 100.2 h).

The phantom was scanned on 18 PET/CT scanners, nine from Philips (Philips Healthcare, Best, The Netherlands) (vendor A) and nine from Siemens (Siemens Medical Solutions, Erlangen, Germany) (vendor B), during a period of 3 days before and 3 days after the reference time (Table 1). Before the calibration, a dedicated ^124^I scan protocol was implemented and tested on each scanner. In the clinical setting, the duration of a whole-body patient scan preferably is limited to 30 min. Therefore, the scan time per axial field of view (FOV) was 2 min for PET/CT scanners of vendor A and 4 min for scanners of vendor B. This difference is a consequence of the difference in length of the effective FOV of the two vendors: ~9 cm for vendor A and ~14–18 cm for vendor B. The standard energy window (approximately 350–680 keV) was applied. All common acquisition corrections were applied, i.e., normalization and corrections for scatter and attenuation, decay, and dead time. If the scanner was EARL-accredited, EARL reconstruction parameters were used [12]. Else, 2D or 3D ordered subset expectation maximization (OSEM) reconstructions with sufficient convergence and a 5-mm full width at half maximum Gaussian reconstruction filter were utilized. If available, the time of flight option on the scanners was applied for acquisitions and in reconstruction.

**Table 1:** Characteristics of included scanners

Site no.	Vendor	ToF	Slices (CT)	EARL^a^	Reconstruction protocol^b^	Voxel size (mm^3^)
1	A	Yes	16	Yes	BLOB-OS-TF	64
2	A	No	16	No	LOR-RAMLA	64
3	A	Yes	16	No	BLOB-OS-TF	64
4	A	Yes	16	Yes	BLOB-OS-TF	64
5	A	Yes	16	Yes	BLOB-OS-TF	64
6	A	Yes	64	No	BLOB-OS-TF	64
7	A	Yes	64	Yes	BLOB-OS-TF	64
8	A	Yes	64	Yes	BLOB-OS-TF	64
9	A	Yes	16	No	BLOB-OS-TF	64
10	B	No	16	Yes	OSEM 2D	14.2
11	B	Yes	40	No	PSF + TOF	49.8
12	B	Yes	64	Yes	PSF + TOF	20.2
13	B	Yes	40	Yes	PSF + TOF	30.4
14	B	Yes	64	Yes	PSF + TOF	11.6
15	B	No	16	No	OSEM 3D	82.9
16	B	No	16	No	OSEM 3D	82.9
17	B	No	40	Yes	OSEM 3D	21.4
18	B	No	40	No	OSEM 3D	49.7

^a^Scanner accredited by EANM Research Ltd (EARL)

^b^Reconstruction protocol name as named by vendor in DICOM header

Two axial FOVs were centered around the vials and scanned subsequently. The start time of the scan was defined as the calibration time (*T*_c_). On each scanner, the phantom was scanned twice; after the first scan, it was turned 180° around the axial axis to determine the reproducibility of the quantification procedure.

**Fig. 2:**
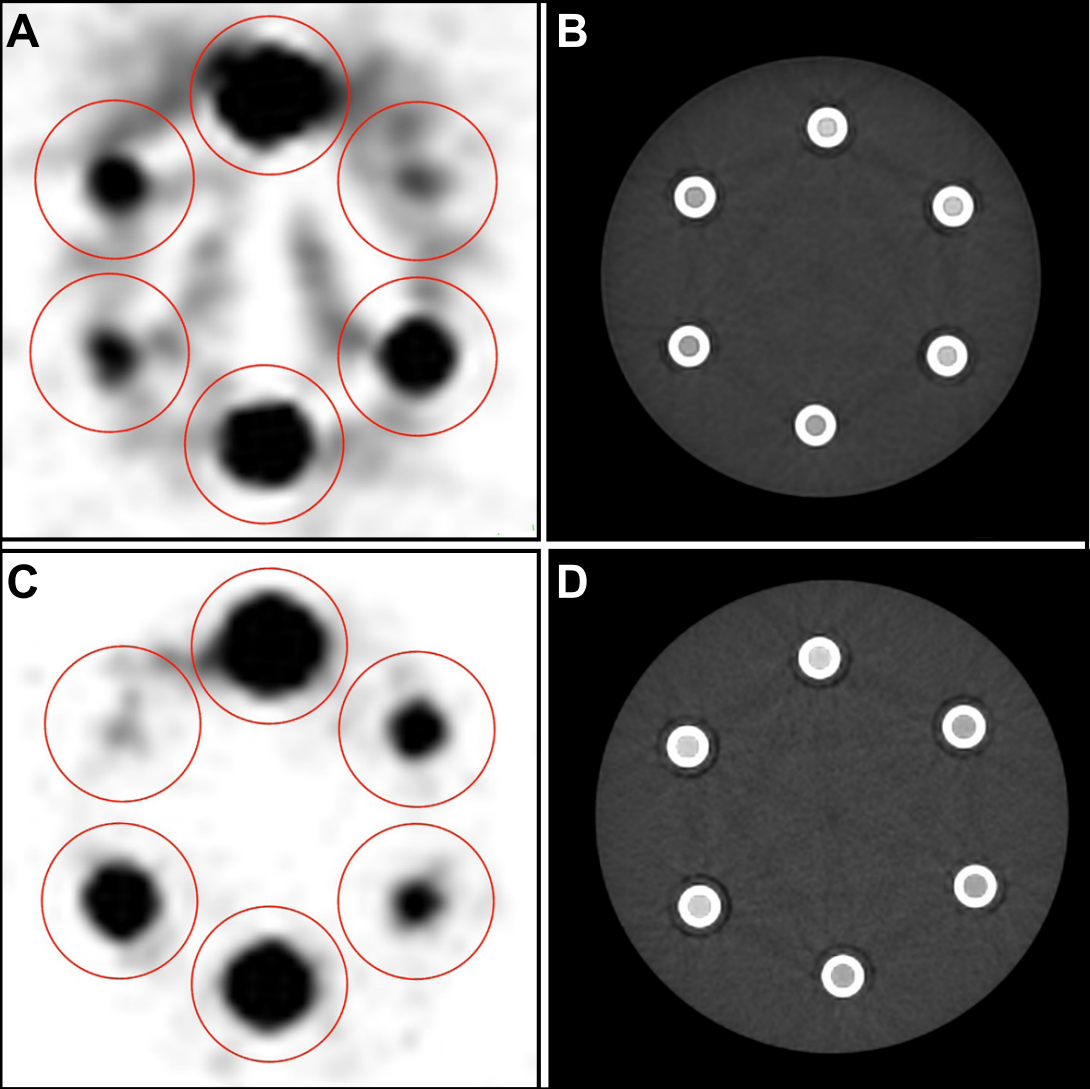
Examples of PET and CT images of axial slices acquired from a scanner of vendor A (**a**, **b**; obtained from scanner number 5) and vendor B (**c**, **d**; obtained from scanner number 15) to emphasize the differences between the two scanner vendors. Both scanners were calibrated shortly after each other, so decay of the reference activities was negligible. At the time of calibration, the top vials contained a reference activity *A*_r_ of 2.49 MBq. Clockwise, the other vials contained 2.6, 127, 514, 13, and 26 kBq, respectively. **a, c** CTAC images, with circular regions of interest matching with the VOIs (in *red*). **b, d** corresponding CT images

A large VOI of 110 mL (46 mm diameter, 66 mm height) was placed around each 2-mL vial (Fig. 2a, d). No interobserver variation was expected and images were analyzed by one person (JK) using the open source software Osirix™ (version 4.1.2, 64 bit) [13]. Measured activities (*A*_m,0_ and *A*_m,180_) were obtained for each acquisition (0 and 180°) and vial.

Per scanner, the data points *A*_m,0_ and *A*_m,180_ were compared with a two-sided paired *t* test. If no significant difference (*p* < 0.05) was obtained, the measurements were considered reproducible, and only *A*_m,0_ was used for further analyses. Data points from *A*_m,0_ were fitted by linear regression to the function *A*_m,0_ = *α*_cal_ + *β*_cal_ · *A*_r_, with and without the additional constraint that the intercept *α* equals zero, so both a proportional and a linear models were investigated. The standard error of the intercept, Δ*α*, was calculated with a two-sided Student’s *t* distribution (*p* < 0.05). If and only if none of the intercepts differed statistically significant from zero, the model without intercept, i.e., *A*_m,0_ = *β*_cal_ · *A*_r_, was applied; otherwise the model with intercept was used (*A*_m,0_ = *α*_cal_ + *β*_cal_ · *A*_r_).

The estimated slope and, if applicable, intercept of each scanner were used to calculate the calibrated activity, *A*_cal_, for each measured activity in each vial, so *A*_cal_ = *A*_m,0,cal_/*β*_cal_ or *A*_cal_ = (*A*_m,0_ − *α*_cal_)/*β*_cal_.

A Bland-Altman analysis was performed for the calibrated activities to assess the relative precision of the scanners. The relative errors between calibrated activity (*A*_cal_) and reference activity (*A*_r_) were calculated for each scanner as an absolute value: *E*_cal_ = │(*A*_r_ − *A*_cal_)/*A*_r_*│.* Subsequently, the measured activities were analyzed with Bland-Altman, again expressed as an absolute value: *E*_m,0_ = │(*A*_r_ − *A*_m,0_)/*A*_r_*│*, to investigate whether calibration improved the measured activities.

All analyses were carried out in Microsoft Excel for Windows, version 2003 (Microsoft Corp., Redmond, WA, USA).

## Results

The phantom was scanned on 18 PET/CT scanners, nine scanners of vendor A and nine of vendor B, in 16 hospitals in The Netherlands (Table 1). Figure 2 shows typical images obtained from scanners of vendor A and B (Fig. 2a, d).

No significant difference between the measured activities *A*_m,0_ and *A*_m,180_ were found in any of the vials of any of the scanners tested.

**Fig. 3:**
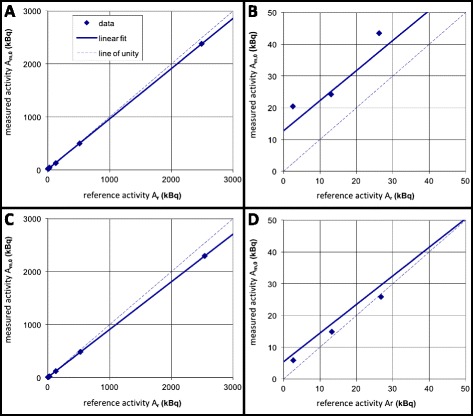
*Graphs* reported to centers of the involved scanners as example of the differences between the scanner vendors. Linear fit of measured and reference activity data according to *A*_m,0_ = *α*_cal_ + *β*_cal_ · *A*_r_. *Top row*: scanner number 5 (vendor A; *α*_cal_ = 13 kBq and *β*_cal_ = 0.947), *bottom row*: scanner number 15 (vendor B; *α*_cal_ = 5.5 kBq and *β*_cal_ = 0.901). Note that the *graphs* on the *right* side (3**a** and **d**) are the same as the corresponding ones on the *left* side (3**a** and **c**), apart from the scales of the axes. Furthermore, the larger offset *α*_cal_ of scanner 5 compared to that of scanner 15 can be foreseen from the larger background signal, shown in **a** and **c**, respectively

The intercepts (*α*_cal_) were significantly different from zero in six out of nine scanners of vendor A (range *α*_cal_ 3–26 kBq, range Δ*α*_cal_ 4–29 kBq). None of the intercepts derived from scanners of vendor B differed significantly from zero (range *α*_cal_ −8–11 kBq, range Δ*α*_cal_ 1–18 kBq). The significant differences between intercept and zero found in six scanners implied the use of the linear model for calibration for all scanners. Applying this model, the *β*_cal_ for scanners of vendor A ranged from 0.80 to 0.98, whereas the *β*_cal_ for scanners of vendor B ranged from 0.85 to 0.97. Two example plots, one scanner of each vendor, of the measured activity (*A*_m,0_) against the reference activity *A*_r_, including fit to the linear model, are displayed in Fig. 3.

**Fig. 4:**
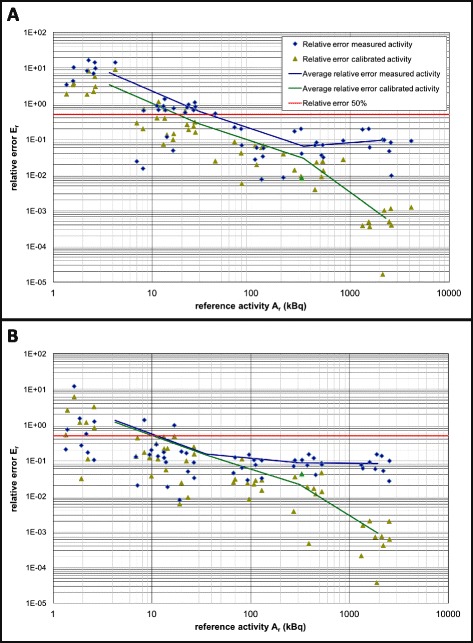
Bland-Altman plots of measured activity *A*_m,0_ and calibrated activity *A*_cal_, versus reference activity *A*_r_ for all scanners, shown per vendor (**a** vendor A and **b** vendor B). For presentation purposes, axes are on a log-log scale, and the relative errors are calculated as an absolute value: *E*_r_ = │(*A*_r_ − *A*_m_)/*A*_r_ *│*and *E*_r_ = │(*A*_r_ − *A*_cal_)/*A*_r_ *│*. To assess improvement of calibration, the average of reference activities *A*_r_ and corresponding average relative errors are calculated for each reference activity decade. The *blue* and *green solid lines* indicate the average relative errors in measured and calibrated activities, respectively

Figure 4 shows the relative errors of the measured activities before calibration (blue data points) and the calculated activities after calibration (green data points). The calibration procedure reduced the relative error in all decades of reference activities. This is depicted by the green and blue line in Fig. 4, indicating the average relative error of the measured and calibrated activities, respectively, for each decade of reference activities. The data in Fig. 4 also indicate in a higher precision the majority of scanners of vendor B. If the relative errors of the measured activities by the scanners are compared to a reasonably chosen threshold of 50 %, Fig. 4 shows that the first scanner of vendor A exceeds 50 % at 43 kBq, while the first scanner of vendor B exceeds this percentage at 16 kBq. After calibration, these activities are 12 kBq (vendor A) and 2.6 kBq (vendor B).

## Discussion

In this study, we present a calibration procedure attaining calibrated scanners for multicenter studies using ^124^I and focusing on DTC and proved it to be feasible. Eighteen PET/CT scanners across the Netherlands were calibrated within 1 week with a convenient and easy to handle phantom, using a single set of ^124^I reference activities and predefined scanner-specific acquisition and reconstruction protocols. The estimated parameters of the applied model were reproducible, making the procedure robust. The calibration resulted in a decrease in relative errors of calibrated activities compared to (non-calibrated) measured activities (Fig. 4), making quantitative data comparable among different centers.

Our relatively simple approach could be used in other clinical multicenter studies focusing on other diseases and tracers, provided that the assumptions of focal uptake and negligible background are fulfilled. This might be the case for other ^124^I tracers and for ^68^Ga, ^18^F, and ^81^Zr tracers.

Standardized and reliable quantification of ^124^I PET imaging is essential in the design of multicenter and dosimetry studies. For example, in a mono-center study by Ho et al., dosimetric analysis of multiple ^124^I PET/CT scans before and after selumetinib treatment of patients with radioiodine refractory metastases was performed. The outcome of this analysis determined whether a new high dose ^131^I treatment would be beneficial [1]. The results of this study are promising; however, in order to expand and validate these, multicenter studies are warranted. This underlines the need for multicenter standardization of scanning and quantification.

The phantom and the procedure were designed with the aim of being reliable, efficient, and safe. Therefore, the design of the phantom used no ^124^I background activity. Although different from previously published calibration procedures for other isotopes [6, 14], the absence of background activity seemed justified from a clinical perspective, and it simplified the calibration procedure significantly. Iodine uptake is highly specific for thyroid tissue and very limited to only a few non-thyroid tissues, like the salivary glands, gastric mucosa, and choroid plexus [15]. These are, however, not in areas of clinical relevance, so uptake in these organs is extraneous for patient image analysis. The main advantage of an empty background was that the partial volume effect could be dealt with straightforwardly. No partial volume correction due to spill in of ^124^I background signal into the signal of the vials was necessary. Additionally, correction for spill out from the vials was possible by drawing relatively large fixed VOIs [16]. By using this method, variable lesion volumes in the phantom were not a requisite. Some practical issues of the calibration procedure could be handled more straightforwardly by omitting background activity. For instance, no solution with a well-known ^124^I background activity had to be produced, making the phantom preparation less complex. Furthermore, legislation for the transportation of the reference activities to all involved centers, as well as for the handling of the activities at the centers, could be met with limited effort due to the lower total activity.

The reference activities used to determine the calibration parameters are traceable to one standard activity. The calibration procedure was designed to support a multicenter study using the same supplier of ^124^I for the study-related patient scans as for the calibration procedure [3, 11]. Since production and activity assessment protocols used by the supplier are standardized, activities used in the calibration procedure and for patient scans in the multicenter study are traceable to a higher standard. In this way, difficulties with activity assessment by dose calibrators as described by Beattie et al. are overcome [17]. However, if in the future multicenter studies using isotopes are produced by more than one supplier, reference activities should be related to a higher, preferably international, primary standard [18]. Potentially, organizations like EARL or EATRIS could play a pivotal role in the development of these standards [12, 19].

The process of dilution and weighing used to produce the reference activities had the risk that an inexact reference activity concentration propagated to the next lower concentration. However, this method is most likely more accurate than that of direct measurement with, e.g., a dose calibrator, due to the low signal-to-noise ratio at low activities [17]. Therefore, this process was preferred, while the risk of propagation of inaccurate concentrations was minimized with an indicative, direct measurement of the produced activity concentrations in each process step.

From the assessment of the precision, it appears that after calibration, the relative error of the scanners of both vendors exceeds the 50 % level at activity levels lower then 2–20 kBq (Fig. 4). It should therefore be kept in mind that if in the clinical setting the uptake is below these lower limits, reliable quantification becomes inaccurate and should not be used for dosimetric calculations. Additionally, this method can be used to assess individual scanner precision in order to exclude underperforming scanners from multicenter studies, provided that multiple measurements per scanner are used.

## Conclusions

A simplified multicenter calibration procedure for ^124^I PET/CT scans in DTC is feasible and results in smaller relative errors in ^124^I quantification. In the future, quantification will be of growing importance especially in multicenter clinical trials, and therefore, standardized calibration procedures need to become applied widely. Our procedure can be used in multicenter ^124^I PET scans focusing on (recurrent) DTC.

## Abbreviations

^18^F-FDG: 2-[18F]fluoro-2-deoxy-d-glucose (^18^F-FDG)
*A*_cal_: calibrated activity
*A*_m_: measured activity
*A*_r_: reference activity
CT: computed tomography
CTAC: CT attenuation corrected
DTC: differentiated thyroid cancer
EARL: EANM Research Ltd
*E*_r_: relative error
FOV: field of view
PET: positron emission tomography
SUV: standardized uptake value
*T*_c_: time of calibration
*T*_r_: reference time
VOI: volume of interest
OSEM: ordered subset expectation maximization
